# Modelling and Detecting Tumour Oxygenation Levels

**DOI:** 10.1371/journal.pone.0038597

**Published:** 2012-06-28

**Authors:** Anne C. Skeldon, Gary Chaffey, David J. B. Lloyd, Vineet Mohan, David A. Bradley, Andrew Nisbet

**Affiliations:** 1 Department of Mathematics, University of Surrey, Guildford, Surrey, United Kingdom; 2 Department of Mathematics, Eidgenössische Technische Hochschule, Zürich, Switzerland; 3 Department of Physics, University of Surrey, Guildford, Surrey, United Kingdom; 4 Department of Medical Physics, Royal Surrey County Hospital, Guildford, Surrey, United Kingdom; University of Arizona, United States of America

## Abstract

Tumours that are low in oxygen (hypoxic) tend to be more aggressive and respond less well to treatment. Knowing the spatial distribution of oxygen within a tumour could therefore play an important role in treatment planning, enabling treatment to be targeted in such a way that higher doses of radiation are given to the more radioresistant tissue. Mapping the spatial distribution of oxygen in vivo is difficult. Radioactive tracers that are sensitive to different levels of oxygen are under development and in the early stages of clinical use. The concentration of these tracer chemicals can be detected via positron emission tomography resulting in a time dependent concentration profile known as a tissue activity curve (TAC). Pharmaco-kinetic models have then been used to deduce oxygen concentration from TACs. Some such models have included the fact that the spatial distribution of oxygen is often highly inhomogeneous and some have not. We show that the oxygen distribution has little impact on the form of a TAC; it is only the mean oxygen concentration that matters. This has significant consequences both in terms of the computational power needed, and in the amount of information that can be deduced from TACs.

## Introduction

The rapid growth that is frequently associated with malignant tumours results in regions of the tumour becoming low in oxygen, in other words, hypoxic. Understanding tumour hypoxia is important because hypoxic cells are both more aggressive and harder to treat [1], [2]. Furthermore, low oxygenation promotes the growth of blood vessels within the tumour (angiogenesis) contributing to the transition from avascular to vascular tumour growth [3]. Yet tissue hypoxia is diffficult to identify in vivo. Invasive techniques, such as the use of an Eppendorf probe, only give local information and can seed cancerous cells along the line of entry.

Non-invasive techniques for the detection of oxygen using positron emission tomography (PET) scans are in the early stages of clinical practice. With PET scanners, a patient is first injected with a radioactive isotope of a molecule that takes a prominent part in whatever process is of interest; most radioactive tracers that are in clinical use focus on the metabolisation of glucose but there are some new tracers, such as [F-18]-flouromisonidazole (Fmiso) and Cu64 diacetyl-bis(N4-methylthiosemicarbazone) (Cu64-ATSM), that are being developed to detect regions of low oxygen concentration. The tracer is distributed around the body by the blood. In the case of glucose detecting tracers, the highest concentrations of the tracer will occur in very active areas, such as tumours. Similarly with Fmiso or Cu64-ATSM the tracer will accumulate in areas of hypoxic tissue. The PET scanner detects the radiation that is emitted from the tracer as it undergoes radioactive decay, and an image of the concentration of the tracer at different parts of the body can then be re-constructed. This re-construction process is difficult resulting in images of relatively poor resolution, typically 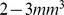. The time dependent decay signal from the PET scanner is known as the tissue activity curve (TAC).

The concentration of the tracer at any location gives a qualitative picture of the degree of tumour hypoxia. Padhani [1] notes that in clinical settings, such qualitative imaging can work well enough, but does introduce a level of subjectivity and that there is a need for greater quantitative understanding. In fact, the concentration of the tracer at any given location is not related to the oxygen concentration of the tissue in a trivial manner and knowing the quantitative relationship between the tracer concentration and tissue oxygenation levels is of great importance if accurate deductions as to the radio-resistance of the tissue are to be made [4]. Indeed, an image created by a snapshot at a single point in time can give a misleading impression because, while for normal tissue the TAC drops after an initial peak, for hypoxic tissue there tends to be a gradual increase in the TAC. This can result in a cross-over point where TACs from both normal and hypoxic tissue give the same result [5] and it is therefore important to consider the TAC at multiple time points. Methods that fit TACs to a nonlinear mathematical model that includes the pharmaco-kinetic behaviour of the tracer and thereby translate the concentration of the tracer to the oxygenation level of the tissue have been developed. The most widely tested of these mathematical models have been compartment models [5]–[8]. These divide the tracer into, typically, three compartments: tracer in the blood plasma; tracer that diffuses freely in the tissue, and tracer that is bound to the tissue via a reaction that is dependent on the concentration of oxygen. The resulting pharmaco-kinetic (PK) models have defined rates of transfer between the different compartments and results in a set of ordinary differential equations that can be solved analytically. The total TAC is a weighted sum of the signal from each of the compartments. The weights and some of the transfer coefficients are calculated by fitting the experimentally determined TACs to the TACs produced by the PK model. The values of the weights and the transfer coefficients are then used to deduce whether the tissue is hypoxic and what kind of hypoxia occurs. Proof of concept experiments have been carried out which demonstrate that PK models have the ability to qualitatively reproduce the features of TACs and distinguish between different types of hypoxia. However, compartment models take no account of the spatial distribution of oxygen. So for PET scan data, the fitting can be done for each individual 2mm^3^ voxel but there is an inherent assumption that the tissue within that voxel is homogeneous and can be represented by an average value. This is not necessarily the case–in vascular tumours, the vessels that deliver oxygen tend to be irregular and tortuous making it likely that the distribution of oxygen within a voxel is highly inhomogeneous. There has been some initial work that includes space explicitly by including tracer diffusion in the tissue and allowing the concentration of oxygen to vary from one point to the next, initially by Kelly and Brady [9] and subsequently by Mönnich *et al* [10]. These studies replace the ordinary differential equation compartment model with partial differential equations. Our original interest was in comparing a partial differential equation model for tracer reactions and diffusion with the analogous compartment model to investigate whether the inhomogeneity of the distribution of blood vessels actually matters on the scale of a voxel. However, this comparison is dependent on first establishing the oxygen distribution within the tissue and that has led to a number of other considerations. In general, if more than a qualitative understanding is to be developed, then one needs to be able to quantify the uncertainties/errors that occur, be they uncertainty that is introduced because of modelling assumptions (for example, whether the tissue can be treated as homogeneous or not), uncertainty due to the difficulty of experimentally measuring parameters that are critical to the model behaviour and finally, computational errors that are introduced due to numerical inaccuracies.

Consequently, the aim of this paper is three-fold. Our first aim is to understand the impact of two particular modelling assumptions. The first relates to the way that oxygen is delivered to tissue. This is a subject that many authors have focussed on and a review article on this subject is given by Goldman [11], yet in even the simplest models of oxygen diffusion and consumption different authors have used different methods and, as we will see, these different assumptions can give quantitatively quite different results. In particular we find that modelling the discrete blood vessels by a ‘source’ term gives a good approximation to the, more realistic, mixed boundary conditions between the vessel walls and the tissue and suggests that efficient algorithms in three-space dimensions could be developed using a source method. The second modelling assumption that we examine is to what extent, on the scale of a voxel, it is important to take account of the spatial distribution of the oxygen in deducing information from TACs, by comparing the results of a partial differential equation model that accounts for oxygen and tracer diffusion with the analogous compartment model.

Our second aim is to examine the sensitivity of the computed oxygenation level of tissue to the various parameters in the model. Measuring physical parameters such as consumption rate of oxygen, diffusivity of oxygen and permeability of blood vessels and the distribution of oxygen is challenging, making it hard to validate any particular mathematical model. However, by understanding the mathematical models one can examine which parameters have a significant effect on predictions that are made by a model and, therefore, which parameters one needs to find for an accurate prediction or, equivalently, to what extent the uncertainty in a particular parameter leads to an uncertainty in the results.

Our final aim is to demonstrate the impact of numerical error that results in the solution of the partial differential equations on too coarse a mesh. We provide computational parameters where the discrete approximations can confidently be considered to be close to the continuous PDE solution.

The paper is outlined as follows. In section 1.1 the simplest models for the diffusion and consumption of oxygen in tissue are re-visited. The different approaches to the different ways of modelling the boundary between the vascular structures that deliver the oxygen and the tissue are examined and a limit is derived in section 1.2 where one expects the mixed boundary conditions used by some authors [12] and the Dirichlet boundary conditions of others [13] to give similar results. In order to separate out the effects of the parameters on the oxygen distribution as compared with how multiple vessels modify the final oxygen distribution we consider a sequence of problems. In section 1.3 we look at just a single vessel and then, we consider a pair of vessels at different distances apart in section 1.4. In section 1.5 we consider multiple vessels and examine to what extent the multivessel results can be understood as a superposition of the single vessel results. In section 2.1 we introduce the particular model of tracer reaction and diffusion that we have studied and examine first the tracer dynamics around a single vessel in section 2.2 and then for multiple vessels in section 2.3, comparing the TACs that results from both random and regular arrangements of vessels. In section 2.4 we fit the TACs produced by the partial differential equation model with a compartment model. In spite of the heterogeneity of the oxygen model we find that the compartment model can distinguish between different levels of oxygen.

## Analysis

### 1. Oxygen Distribution

Many mathematical models of oxygen transport are built on the Krogh-Erlang cylinder model [14] that models oxygen transport by a diffusive process through a homogeneous medium governed by the equation
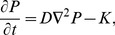
(1)for the oxygen partial pressure 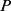 within the tissue, where 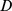 is the diffusivity of oxygen in tissue and 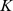 describes oxygen consumption by the tissue. In the original Krogh-Erlang model [14], the oxygen partial pressure was fixed at the vessel wall (a Dirichlet boundary condition) with the consumption rate 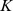 set to be constant. This latter assumption means that equation (1) has to be supplemented with a requirement that the consumption rate is zero when 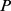 is zero to prevent the equations from giving solutions with regions of negative partial pressures. A more realistic form for the oxygen consumption term in equation (1) is the Michaelis-Menten form,
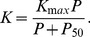
(2)With this nonlinear consumption rate, as 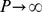 the consumption asymptotes to the constant value 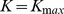, so that when oxygen is abundant, consumption is approximately constant. However, when oxygen is scarce, oxygen consumption is proportional to the amount of oxygen available. This choice for 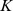 means that the oxygen partial pressure remains positive (or zero) at all times.

In Goldman [11] all the underlying assumptions of the Krogh-Erlang cylinder model are listed and a thorough review of current work that relaxes these assumptions is given. Of particular relevance here is the intravascular 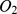 resistance (IVR); in the original Krogh-Erlang model the use of Dirichlet boundary conditions at the vessel wall excluded the possibility that the oxygen delivery to the tissue may be dependent on the partial pressure difference across the vessel wall. As discussed further below, this is only valid if the vessel wall is sufficiently permeable to oxygen.

**Figure 1:**
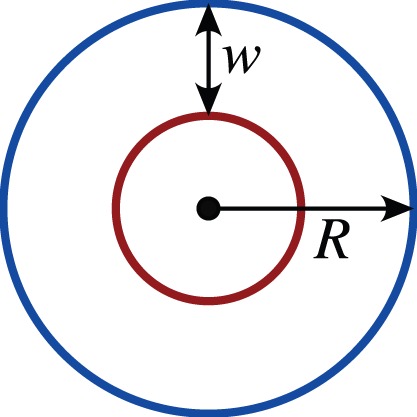
Cross-section of a single vessel with a wall.

One way of including IVR is to ignore intravascular processes but to model the flux of oxygen as it diffuses across the vessel wall, and then on into tissue explicitly via a mixed boundary condition, sometimes known as a Robbins boundary condition. This mixed boundary condition arises as follows: assuming that a blood vessel wall consists of two concentric cylinders of outer radius 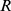 with width 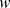 between the two cylinders, as shown in cross-section in figure 1, and that there is just diffusion and no consumption by the wall tissue, then the flux at 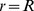, 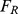 is given by
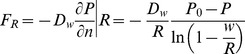
(3)where 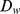 is the diffusivity in the wall and 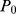 is the partial pressure of oxygen inside the vessel. For capillaries 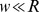, (typical vessel radii are 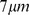 [12] and vessel walls are 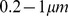 [9]) and equation (3) becomes
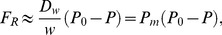
(4)where 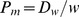 is the permeability of the vessel. The inclusion of IVR can therefore be modelled by using the boundary condition (4) at the vessel wall.

An alternative model that includes IVR is to model the vessels by a so-called distributed source where instead of modelling the vessels as discrete entities leading to the solution of the diffusion equation on a punctured domain, the source is represented by a function which has localised spikes at the vessel positions [9]. With such a source term, the diffusion model becomes
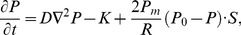
(5)where 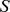 is referred to as the vascular map and is a function that takes the value 1 for regions inside the vessel and 0 otherwise. This is a modification of a term that was first introduced by Baxter and Jain [15] for modelling tumours at the whole tumour scale. The motivation for the particular form for the source term comes from considering the flux across a membrane as in equation (4). Then the net rate of oxygen diffusing for an individual blood vessel per unit volume is given by
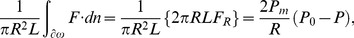
(6)where 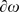 is the surface of the blood vessel. So the diffusion model (1) then becomes equation (5).

The derivation of equation (4) and subsequently equation (6) assume that oxygen within the blood vessel is well-mixed and that consequently the partial pressure at the interior of the vessel is fixed at 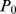. Detailed earlier work by Hellums and co-workers has shown that IVR actually arises as a consequence of the way that oxygen is transported and released by red blood cells [16], [17]. Hellums *et al* [17] showed that the delivery of oxygen to tissue could be described well by a flux of the same form as equation (6), where 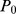 is the partial pressure in the vessel corresponding to the mean haemoglobin saturation.

Some studies have included IVR [18] and some have not [13], but there has been no systematic comparison of the two. Likewise, although some authors have used source terms [9], [12] and some have used models that describe capillaries as discrete entities there has been no comparison of these two methods. This is relevant because, Dirichlet boundary conditions may sometimes be used for the pragmatic reason that they are easier to code but, in fact, can only be justified in the situation that the permeability of the wall is sufficiently high. Similarly, there are computational advantages to having a domain that is simply connected, as occurs if the source term formulation is used. A number of studies have investigated oxygen diffusion in three space dimensions [18], [19]. However, the difficultly in correctly implementing the vascular structure and the high computational cost of such simulations mean that it is valuable to thoroughly examine the modelling issues relating to the boundary conditions at the vessel wall in two space-dimensions before considering the three-dimensional problem.

In the rest of this section a quantity that determines whether Dirichlet boundary conditions are appropriate is derived. Then, in order to examine which parameters are significant in determining the level of oxygenation we non-dimensionalise the equations and consider a sequence of problems: first considering the impact of the parameters on the oxygen distribution created by a single vessel and then examining how a pair of vessels interact before, finally, considering tissue with realistic vascular structures.

#### 1.1 Mixed versus Dirichlet boundary conditions

If a boundary is sufficiently “leaky”, one would expect mixed and Dirichlet boundary conditions to give the same results. An idea for what is “sufficiently leaky” can be obtained by considering steady-states of equation (1) for a single vessel which satisfy
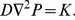
(7)


For a cylindrical vessel with no axial dependence, this reduces to
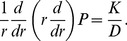
(8)


In case that 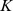 is constant, equation (8) is exactly soluble and gives
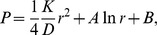
where 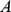 and 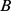 are integration constants. At some radius 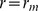 the oxygen partial pressure will drop to zero and there will be no flux of oxygen. Applying the boundary conditions 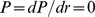 at  gives



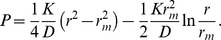



The maximum oxygen diffusion distance, 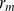, is determined by the boundary condition at 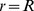. Using 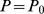 at 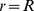 leads to the equation
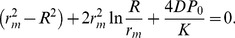
(9)


Using the mixed boundary condition gives
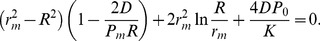
(10)


As 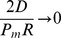 equations (9) and (10) for 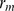 become identical, suggesting that provided 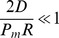 both mixed and Dirichlet boundary conditions will give similar results. Typical values for 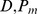 and 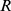 for tumour blood vessels (see table S1). result in a value for 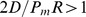, suggesting that Dirichlet boundary conditions are unlikely to give similar results to mixed boundary conditions.

#### 1.2 Non-dimensionalisation

The original problem has six parameters describing tissue and vessel properties, namely, the tissue consumption parameters 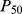 and 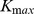, the oxygen diffusivity 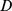, the permeability of the blood vessel to oxygen 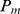, the partial pressure of the oxygen within the blood vessel 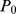 and the vessel radius, 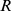. The process of non-dimensionalisation shows that the six tissue and vessel parameters are not truly independent, and the problem can be reduced to just three non-dimensional parameters namely, the scaled partial pressure inside the vessel 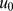; the scaled permeability 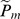 and the scaled vessel radius, 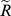. The advantage of studying the non-dimensionalised equations is that one has a much reduced parameter space to investigate.

The equations are rescaled by defining 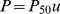 and scaling the length by 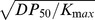. Consequently, for the steady-state solution of the reaction-diffusion equation with rate given by equation (2) three different problems are considered: (i) Dirichlet boundary conditions, (ii) mixed boundary conditions, (iii) distributed source term. These are listed below.

For **Dirichlet** boundary conditions:
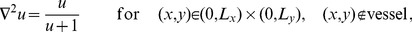
(11)

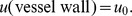



For **mixed** boundary conditions:
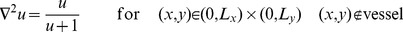
(12)

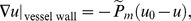
where 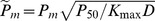.

For **the source** term:
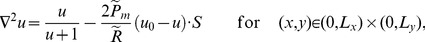
(13)where
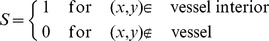
Typical values for the measured physical parameters are listed in table S1 and the corresponding ranges of values for the non-dimensional parameters are given in table S2.

#### 1.3 Computations for a single vessel

With a single vessel the diffusion problem is axi-symmetric and the diffusion problem in two-space dimensions can be reduced to a diffusion problem in one, radial, direction. For example, equation (12) becomes
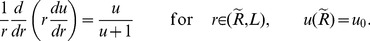
(14)and the source case becomes
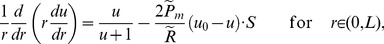
(15)where



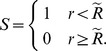



**Figure 2:**
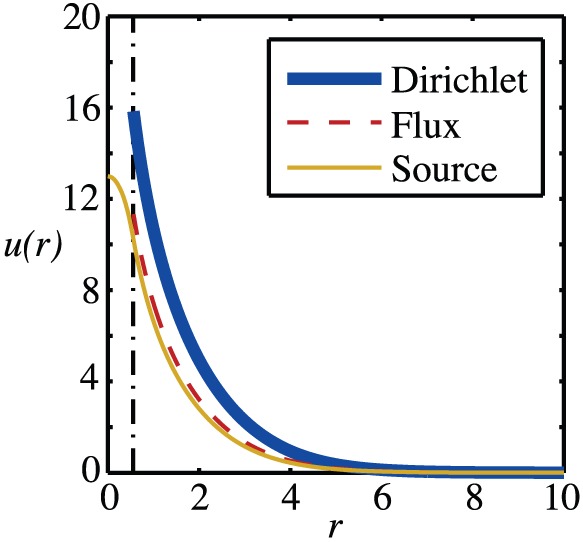
Typical solutions for the oxygen profiles from a single vessel for different boundary conditions. Parameter values are 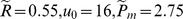.

Each case leads to a boundary-value problem. For the flux and Dirichlet cases this problem was solved on the large but finite domain, 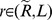, corresponding to the region outside a vessel of radius 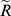. The value for 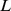 was chosen sufficiently large (typically 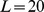) that the results were independent of whether Dirichlet or Neumann boundary conditions were chosen at 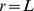. In the cases of the source term, 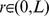 with 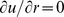 at the lefthand boundary. Each one-dimensional problem was solved using the matlab boundary value problem solver bvp4c; typical solutions are shown in figure 2. All boundary conditions lead to a simlar, monotonically decreasing, profile: in fact the maximum principle can be used to show that the maximum value of the oxygen has to occur on the boundary. The difference between the various boundary conditions is that with Dirichlet boundary conditions, this maximum value is pinned to the value of 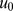, the scaled partial pressure in the blood vessel, but in all other cases the maximum value is at some value that is lower than this. The consequence of this pinning of the partial pressure to the value of 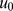 at the vessel boundary is that the Dirichlet boundary conditions tend to give higher levels of oxygenation than mixed boundary conditions or the source term. On figure 2 the vertical dashed line represents the boundary of the vessel. The ‘maximum diffusion distance’ for oxygen in tissue is often quoted as 70 microns, equating to approximately 4 units in the non-dimensional units used in figure 2. Considering just a single vessel with a Michelis-Menten consumption term, there is no maximum diffusion distance for oxygen in that the oxygen decreases monotonically with distance from the vessel, with 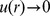 only as 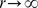. The ‘maximum diffusion distance’ can therefore only be specified in terms of a distance below which the level of oxygen is too small to be detected.

In order to compare the different solutions systematically as the parameters are varied, we have considered two different measures. Firstly the the L^1^ norm, 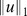, where
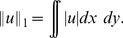
(16)


The L^1^ norm is related to the average level of oxygenation, 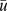, of a piece of tissue of area 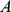 by
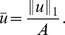
Oxygenation of tissue samples on the microscale are often examined using tissue staining where a dye that is oxygen sensitive is applied to a tissue slice. This tends to lead to a binary measure: either oxygen is present or not (or only at a concentration below a threshold value). Results are often quoted as a hypoxic fraction, that is the fraction of the tissue that is hypoxic. In order to mimic this kind of measure we have also calculated the ‘oxygenated area’, 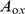, which is the area for which the oxygen partial pressure is greater than a threshold value 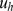


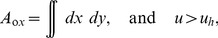
(17)For a given area of tissue 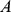 the hypoxic fraction 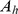 could then be calculated from



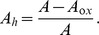
As can be seen from figure 2, the calculated value for the oxygenated area will depend strongly on the particular value of the threshold 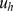 that is chosen: for the Dirichlet boundary case depicted in figure 2, threshold values 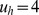 and 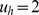 lead to oxygenated radii of 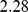 and 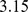 respectively. In turn, these values lead to oxygenated areas of 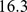 for 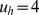 and nearly double that value, 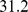 for 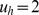. Different threshold values are important for different aspects of a cell function, but broadly oxygen levels below  (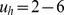, for typical parameter values) have a significant impact on the outcome of cancer therapies [1]. In the case of radiation treatment, half-maximal sensitivity to radiation therapy occurs at oxygen levels of 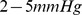 (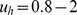). Typical tissue staining techniques stain tissue at threshold values of between 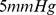 and 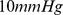 (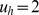 and 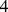). Given the sensitivity of the results to the value of the threshold, if comparison is to be made with experimental data, it is particularly important that an accurate value for this threshold is known and this in itself can be difficult. In Pogue *et al* [12] a careful study fitting a diffusion model for oxygen with vascular maps derived from real tissue samples was performed. They found their results were very sensitive to the threshold that was chosen and that their model fitted the data best for a value for the threshold that was much lower than the conventionally accepted value. For many of the results that are presented in this paper, we have selected the value 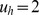.

**Figure 3:**
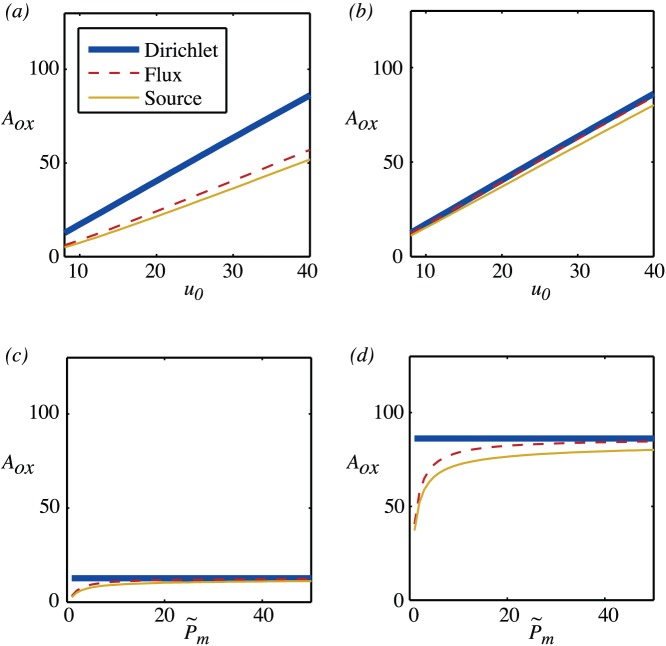
Oxygenated area for a single vessel with non-dimensional radius 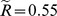 and 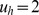. (a) Fixed permeability, 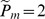 and varying vessel partial pressure, 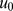. (b) Fixed permeability, 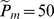 and varying vessel partial pressure, 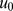. (c) Fixed vessel partial pressure, 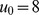 and varying permeability, 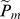. (d) Fixed vessel partial pressure, 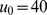 and varying permeability, 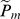.

Results for the oxygenated area for fixed radius but varying vessel partial pressure 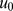 and permeability 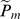 are shown in figure 3. As is to be expected, these show that at high permeability, all three sets of boundary conditions give similar results. At low permeability, the source term representation gives a reasonable approximation to the mixed boundary conditions. Note that the condition found in 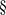
?? for the Dirichlet and flux boundary conditions to coincide translates to 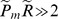 or, for 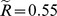, 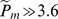. For the experimentally measured range of values of 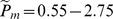, figure 3(c) and (d) show that modelling oxygenation using Dirichlet rather than flux boundary conditions will result in an over estimate for the oxygenated area and that this is more significant the lower the vessel partial pressure. So, for example, for the low scaled vessel partial pressure of 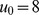 (equivalent to 20mmHg), mixed boundary conditions give an oxygenated area of 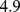 and Dirichlet boundary conditions give a value that is more than two and a half times bigger of 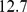. Even at the highest scaled vessel partial pressures, 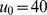 (equivalent to 100mmHg), mixed boundary conditions give an oxygenated area of 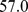 and Dirichlet boundary conditions give a 50% larger value of 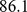.

**Figure 4:**
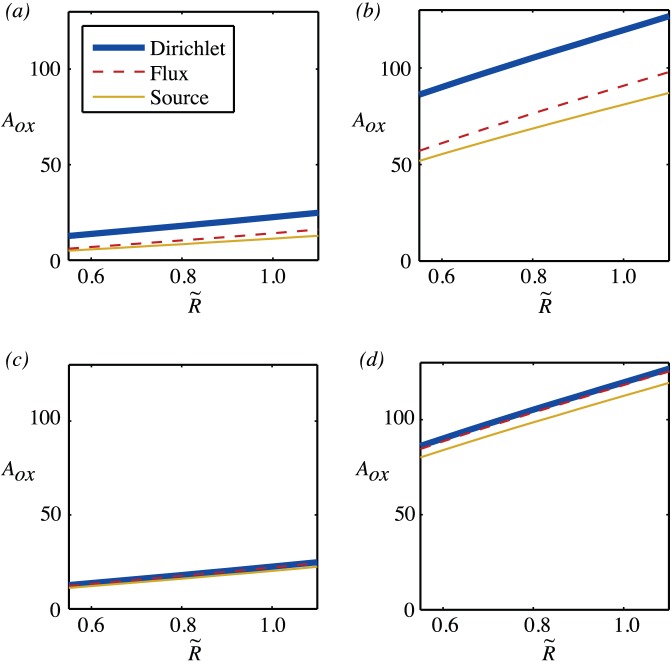
Oxygenated area of a single vessel for 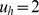 and varying vessel radius, 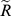. (a) 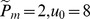. (b) 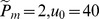. (c) 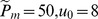. (d) 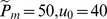.

In figure 4 the oxygenated area for varying scaled vessel radius is shown. These show that the oxygenated area is approximately linearly related to the scaled vessel radius.

Together, figures 3 and 4 show that, for the typical ranges of permeability that are quoted for blood vessels, it is important to take account of the IVR and that either using a mixed boundary condition or a source term will give similar results. The oxygenated area is sensitive to the scaled vessel partial pressure and to the scaled permeability and an uncertainty of 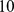
% in either of these values will lead to a similar order of uncertainty in the oxygenated area. The oxgyenated area is much less sensitive to the scaled vessel radius.

#### 1.4 Two vessels

**Figure 5:**
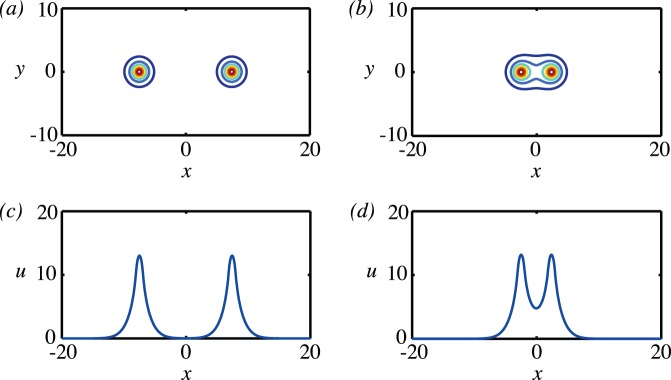
Contour plots and solution profiles for two vessels placed at different separations. In all cases, 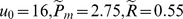 and the source model for the delivery of oxygen to the tissue was used. (a) Contour plot for two widely separated vessels, seperation 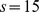. (b) Contour plot for two vessels that are close enough to interact, separation 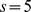. (c) Oxygen concentration profile along the line 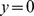 for 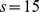. (d) Oxygen concentration profile along the line 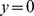 for 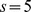.

In a piece of tissue there is typically many vessels, not just a single isolated one. If two vessels are sufficiently far apart, then each will be unaffected by the presence of the other, as illustrated in figure 5(a) and (c). As they become closer and closer, the oxygen distribution around each vessel will become more and more affected by its neighbour, see figure 5(b) and (d).

The computations shown in figure 5 were carried out on a two-dimensional domain with a grid of 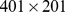 grid points using a square mesh of grid size 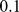. Grid refinement checks were carried out to check that this was sufficiently fine (see table 1). The grid refinement checks suggest that, for results that are accurate to 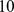
%, a grid of between two and four grid points per vessel should be used. We note that in order to minimize computational cost, previous studies have frequently used a grid of spacing of the same size as the vessel and that this will introduce an error of 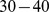
%, depending on the type of boundary conditions used.

**Table 1:** Grid refinement check.

Grid spacing	grid/vessel ratio	Dirichlet 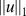	Source 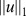
1.0000	1	1089.671	818.8468
0.5000	2	1716.200	1270.952
0.2500	4	1641.403	1036.248
0.1250	8	1774.854	1146.761
0.0625	16	1808.991	1141.513
0.0400	25	1831.189	1147.694

The L^1^ norm as a function of grid size for two vessels separated by 5 units with 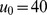 and 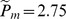.

**Figure 6:**
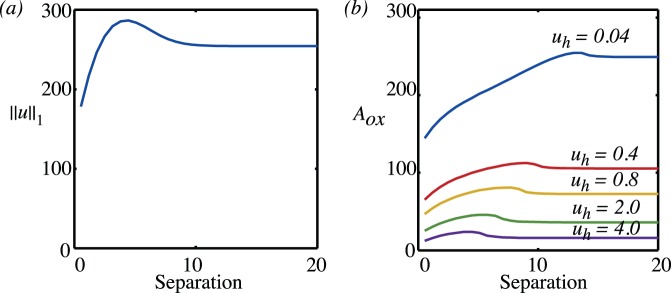
(a) 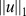 norm and (b) oxygenated area as a function of separation. Different vessel partial pressures are shown corresponding to, 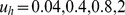 and 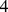 (typically corresponding to 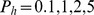 and 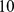 mmHg). The source term model for oxygen delivery has been used; the behaviour for Dirichlet or flux boundary conditions is qualitatively similar. Each vessel has a scaled radius 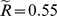 and scaled permeability 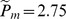. The scaled vessel partial pressure is 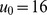 (typically corresponding to 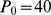 mmHg).

We have systematically examined how the 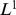 norm and the oxygenated area vary as the separation between the vessels is changed and the results are summarised in figure 6 as a function of the separation. Only the oxygenated area is shown as the results for the 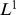 norm are qualitatively similar. For vessels sufficiently far apart, the 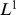 norm and the oxygenated area are twice the values calculated in 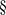
?? for one vessel. This corresponds to the flat section to the far right of figure 6 and shows that for a separation 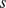 greater than about ten the vessels interact only minimally. Note that in these non-dimensional units, this represents a separation of approximately nine vessel diameters.

As the vessels get closer, the oxygenation of the tissue initially increases but then decreases approaching the value of the oxygenation produced by a single vessel as 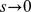. The increase is much more prominent in the 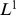 norm than in the oxygenated area, reflecting the fact that the dominant effect of two vessels close together is that the level of oxygenation of the oxygenated tissue increases, rather than that more tissue reaches the oxygen threshold value of 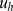.

#### 1.5 Multi-vessel

In normal tissue, blood vessels are regularly spaced in order to deliver oxygen to tissue in an optimal manner. In tumour tissue, this is not the case and the position of blood vessels is much more closely represented by a random distribution, resulting in a log-normal distribution for the distance between blood vessels.

**Figure 7:**
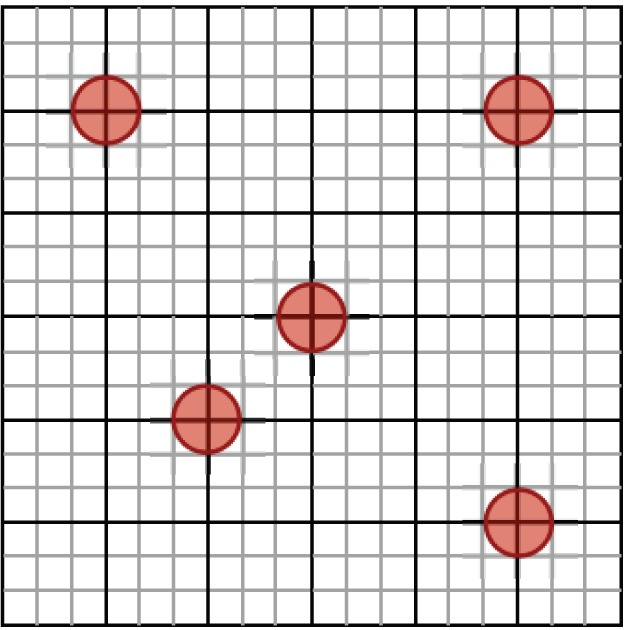
Vascular grid versus computational grid. Blood vessels are located randomly on a fixed coarse vascular grid (solid black lines) allowing a refined computational grid (light grey lines).

First we outline how we distribute vessels on the plane while still being able to carry out computational grid refinements. In order to randomly place the vessels without overlap we first construct a ‘vascular grid’ that has a grid length of 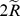. Vessels are placed so that their centres are at random points of the vascular grid. The choice of grid length means that no vessels can overlap each other. A computational mesh is then constructed that forms a sub-grid of the larger vascular grid, one example is shown in figure 7. This computational mesh can be refined to give adequate numerical resolution. Computations were carried out on a domain of 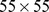 in non-dimensional units, equating to 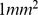 of tissue for typical values of the parameters. As for the two vessel case, a grid spacing of 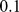 gave good resolution. The effect of varying microvessel density (MVD), was considered by solving equations (13) for a sequence of different MVDs. For each value of the MVD, ten different random vascular maps were created and the L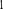 norm and the oxygenated area calculated. The random selection of points on the vascular grid results in vascular maps which have a log normal distribution of nearest neighbour distances. As the MVD increases, both the mean and the variance of this distribution decreases (mean 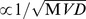, variance 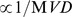). We have considered MVDs in the range 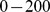 per mm^2^, which includes the values used in previous studies of tumour oxygenation [9], [13].

**Figure 8:**
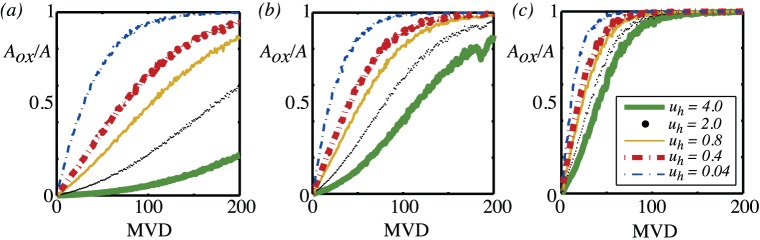
Mean of ten realisations of the oxygenated area versus vessel density (MVD). (a) 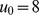 (b) 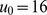 c) 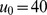. In all cases, 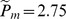 and the source model for oxygen entry from the blood vessels is used. The different lines on each plot represent different values of the threshold, 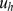 used to measure the level of oxygenation.

Commonly quoted values for vessel partial pressures range from 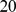
mmHg to 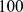
mmHg where, 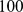
mmHg is typical of arterioles and 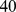
mmHg typical of venules. Often tumour supply is from the venule side, and although some of the results that are presented below are for the full range from 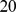
mmHg to 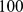
mmHg (8 to 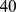 in nondimensional units), more detailed results are shown for the case of vessel partial pressure 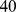
mmHg (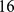 in nondimensional units). The results for the fraction of the area of the tissue that is oxygenated for three different values of the vessel partial pressure 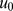 and varying hypoxic levels 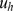 are shown in figure 8. The general trends are not surprising: more vessels are needed to oxygenate more tissue up to some cut-off number beyond which all the tissue is oxygenated; the vessel density that is needed for the tissue to be oxygenated depends on the value that is used to specify oxygenation (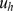), with more vessels needed the higher the value of 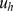. Typically, tissue is considered to be hypoxic if it has partial pressures in the range 1-10mmHg and necrotic for partial pressures less than 1mmHg. So, for example, in figure 8(b) corresponding to vessel partial pressures of 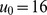 (40mmHg) and for a micro-vessel density of 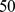 vessels/mm^2^, typically 90% of the tissue receives some level of oxygen, but for most of the tissue this is at too low a level to be significant resulting in the fact that only approximately 15% is oxygenated, 35% is hypoxic and the remaining 50% is necrotic.

**Figure 9:**
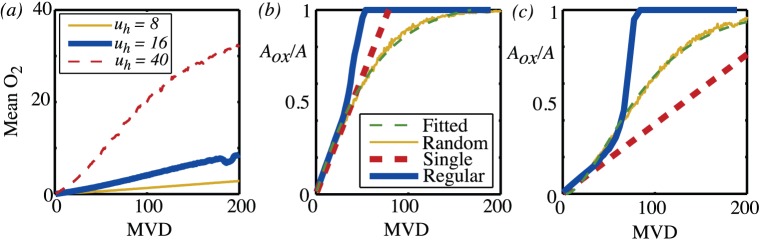
Mean tissue oxygenation and oxygenated area. (a) The mean oxygen level for three different values of the vessel partial pressure. (b) and (c) The fraction of area that is oxygenated for 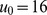 using 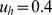 in the case of (b) and 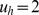 in the case of (c).

The computational cost of simulating multi-vessel distributions to attain average quantities leads one to ask whether one could predict the multi-vessel results just from the one-vessel results. In particular, if the vessel density is low, one might expect the oxygenated area of the multi-vessel distribution to simply be the oxygenated area given by a single vessel multiplied by the number of vessels, i.e.
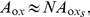
(18)where 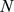 is the number of vessels and 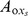 is the oxygenated area of a single vessel. Figure 9 shows the oxygenated area for two different values of 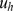 in more detail and compares the results with a number of approximations. We focus on the value of scaled vessel partial pressure 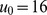 (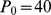
mmHg for typical parameter values) since this is the most widely quoted value for the vessel partial pressure in tumour tissue. For microvessel densities up to around 50 mm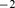, the vessels do not interact, and the approximation give by equation (18), the dashed line in the figure, works well. That the vessels do not interact is further underlined by the fact that up to a MVD of around 50, there is no difference in the oxygenated area produced by a regular grid of vessels (shown by the thick line in figure 9) and that produced by a random arrangement of vessels.

### 2. Tracer

Having discussed some aspects of modelling oxygen diffusion in tissue, we now consider the issues with trying to detect that oxygen using a tracer. First we solve a model for tracer reaction and diffusion that includes the spatial distribution of oxygen. We then examine to what extent a compartment model for tracer dynamics can accurately determine the level of oxygenation.

#### 2.1 Modelling and non-dimensionalisation

The detection of oxygenation via PET scanning techniques first requires a radioactive tracer to be injected into the blood. The tracer is transported by the blood: some is removed from the blood by the kidneys and some diffuses into other body tissues. Once in the tissue, some of the tracer will bind at a rate that is dependent on the local oxygen concentration with the tracer being bound more effectively at low oxygen levels. A detailed pharmacokinetic study of the binding process for FMISO was carried out by Casciari *et al* [6]. Although their compartment model did not allow for any spatial variation, nevertheless they were able to show that the model could replicate typical behaviour of TACs from both a more complicated, but still spatially homogeneous, model and clinically extracted TACs. They did find that including some transport limitations into the compartments representing tracer in the tissue was important.

In order to take account of the diffusion of tracer and the spatial distribution of oxgyen, Kelly and Brady [9] suggest taking the model for the partial pressure of oxgyen, equation (5) and coupling it to a partial differential equation for the tracer,
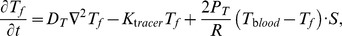


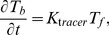
(19)where 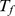 is tracer that is free to diffuse and 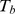 is bound tracer. The parameter 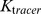 is the rate at which the free tracer is bound and is dependent on the oxygen partial pressure 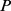,



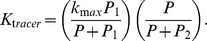



The second term in brackets acts as a switch to turn off the binding if tissue is necrotic. The concentration of tracer in the blood, also known as the plasma input function, 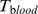, is modelled as the sum of two exponential decays,
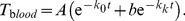
(20)


The first term represents the dispersal of the tracer around the body, the second the renal term representing the removal of tracer by the kidneys. Typically, 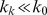. Implicit in modelling the tracer in the blood in this way is that the tumour is a small volume compared with the volume of the rest of the body. Consequently the fact that some tracer flows into the tumour has a negligible impact on 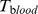.

Mönnich *et al* use a similar model [10], but instead of using a source term to model the entry of tracer from the blood they use mixed boundary conditions. In section 1 we found that for oxygen diffusion using a source term gave comparable results to the use of mixed boundary conditions, and we would expect this to be the same for the tracer. Kelly and Brady [9] show that this model can reproduce typical TACs by considering random distributions of vessels. Mönnich *et al* do a similar comparison, but this time using vascular maps that are obtained from tissue staining. Comparing with experimentally determined TACs shows that the partial differential equation model does mimic the behaviour that is seen in practice. Our aim here is to examine to what extent the partial differential equation (19) model is needed in order to accurately model the behaviour that is seen and to what extent a compartment model is adequate.

As for the oxygen problem, we first non-dimensionalise by rescaling space by 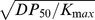 and the oxygen partial pressure 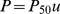 and introducing 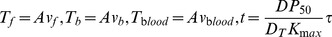. This results in the model for the oxygen and tracer as:
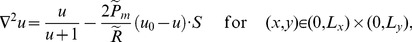
(21)and for free and bound tracer,



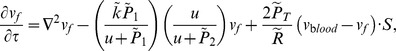


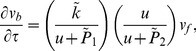
(22)where the scaled plasma input function is
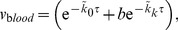
(23)and
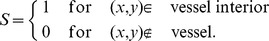
Since at the start there is no tracer in the tissue, only in the blood plasma, both 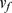 and 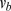 are set to zero initially.

The scaled parameters for the tracer dynamics are 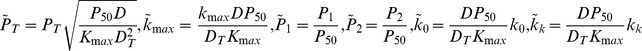. The process of non-dimensionalisation reduces the original 17 parameters to 10.

#### 2.2 Single vessel

**Figure 10:**
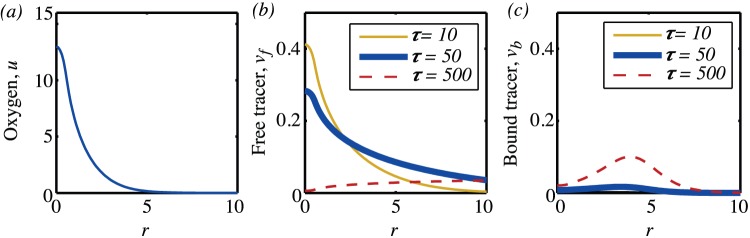
Oxygen and tracer distributions in space. (a) Oxygen partial pressure against 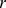. (b) Free tracer against 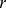 for three different times. (c) Bound tracer against 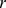 for three different times. The parameters are: 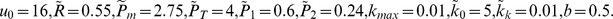

Before considering how randomly distributed vessels within a piece of tissue behave, we first examine a single vessel. Equations (21) are solved to find the steady-state oxygen distribution as shown in figure 10(a). Then, equations (22) are solved to give the concentrations of free and bound tracer as a function of time and space. For these computations and for those in the following sections we have chosen representative values from the literature for the various parameter values. The parameter ranges are given in in table S1 for the dimensioned parameters and in table S2 for the corresponding non-dimensional parameters.

Typical concentrations of the free and bound tracer as are shown in 10(b) and (c) respectively for three different points in time. These show how, initially, the dominant effect is the diffusion of the tracer into the tissue–at 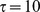 there is essentially no bound tracer but tracer has diffused a considerable distance from the vessel (note the vessel radius is 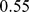 in these non-dimensional units). However, as time goes on the binding process becomes important–by 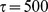 the way that the binding is dependent on the oxygen level is apparent, with both the low binding rate at high oxygen concentrations and the effect of the necrotic term resulting in the shape of the bound oxygen profile in 10(c). In fact, the maximal binding rate occurs when 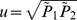 which, for the parameters we have used is 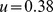.

**Figure 11:**
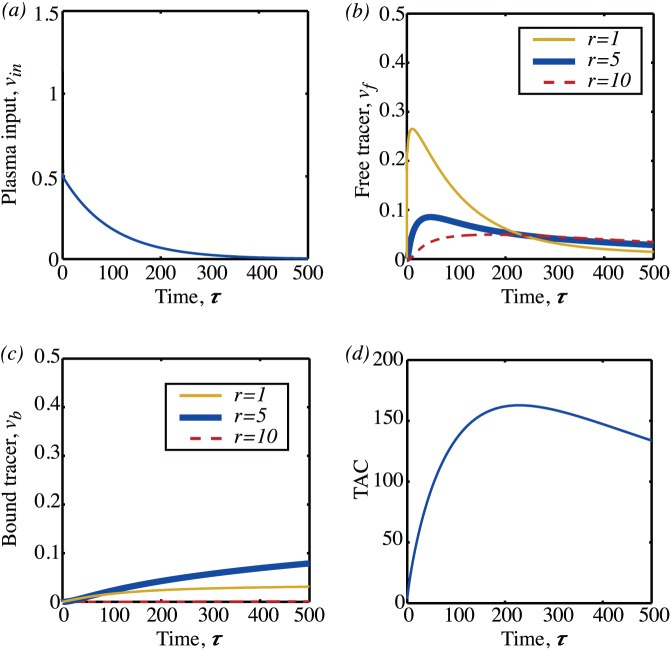
Plasma input function and tracer time evolution. (a) Plasma input function. (b) Free tracer against time for three different points in space. (c) Bound tracer against time for three different points in space. (d) Tissue activity curve.

In order to further illustrate the behaviour, in figure 11(a) the decay of the plasma input function, equation (23), as a function of time is shown and, in figure 11(b) and (c), the concentration of free and bound tracer respectively are shown for three different points in space. For the parameters we have chosen the initial concentration of the plasma in the blood vessel is 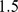. However as 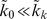 this value rapidly drops, so fast that on the timescale shown this is not visible, and in fact the blood plasma term is dominated by the second term in equation (23). As time continues, the free tracer diffuses from the blood vessels into the tissue, so that at any particular location the free tracer concentration initially increases with time, as seen in figure 11(b). The further from the blood vessel, the longer it takes the tracer to diffuse, so the slower this increase. At the same time as diffusing in space, the free tracer binds at a rate dependent on the oxygen, and this ultimately leads to the decay of the free tracer over time. In figure 11 (c) the growth of the bound tracer as a function of time for three different points are shown. At 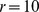, the bound tracer is zero because the oxygen concentration is so low that the tissue is necrotic.

A TAC consists of the sum of signals from the plasma, free and bound tracer,
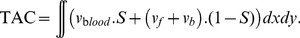
(24)


The general characteristics of TACs from normoxic, hypoxic and necrotic tissue can be seen by considering the three points 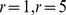 and 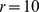 respectively, as shown in figure 11. For 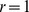, the sum of the free and bound tracer will show a very rapid increase from zero initially to a high level and then a slower but still fairly rapid decline. Whereas, for 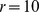, the tissue is necrotic and there is effectively no bound tracer. The only tracer contribution to the TAC then comes from the free component, which because of the distance of this point from the blood vessel, shows only a gradual increase that is diffusion limited. The point 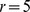 sits between these two extremes. That there is a cross-over point, as mentioned by Wang *et al* [5], where both oxygenated and hypoxic tissue would give the same image is clearly seen.

#### 2.3 Multi-vessels

**Figure 12:**
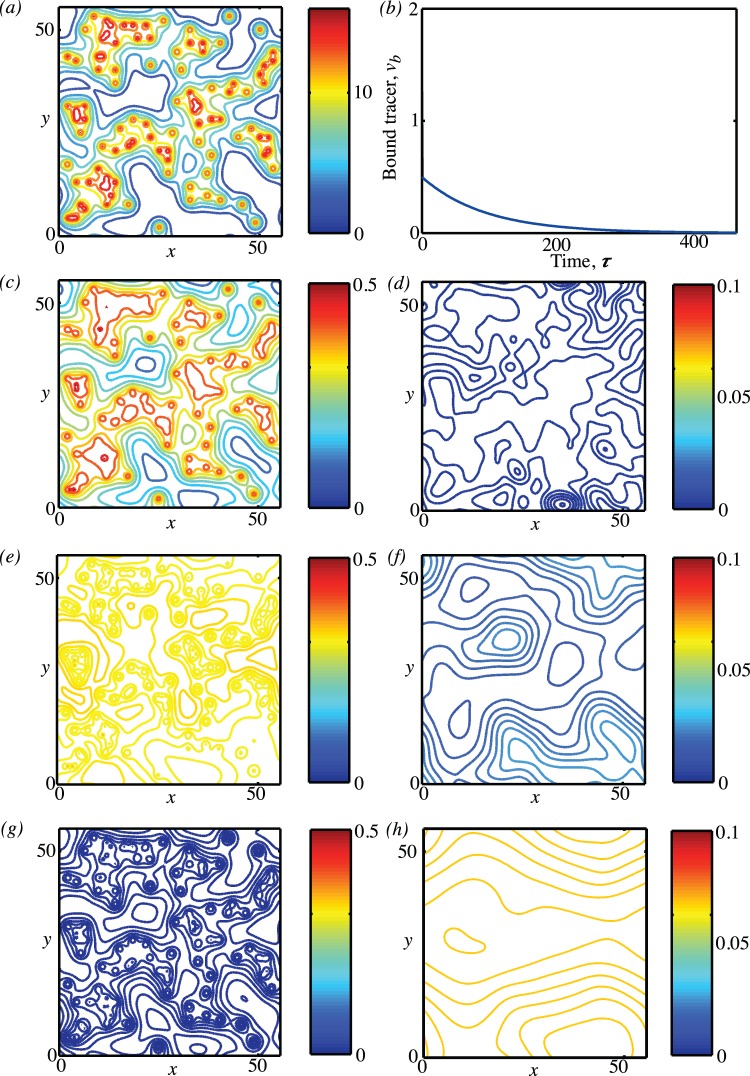
Oxygen map and contours of free and bound tracer as a function of time. A microvessel density of 100 mm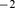 has been used. (a) Oxygen concentration. (b) Plasma input function as a function of time. (c) 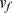 at 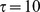. (d) 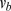 at 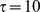. (e) 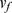 at 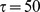. (f) 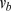 at 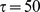. (g) 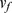 at 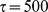. (h) 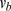 at 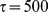.

**Figure 13:**
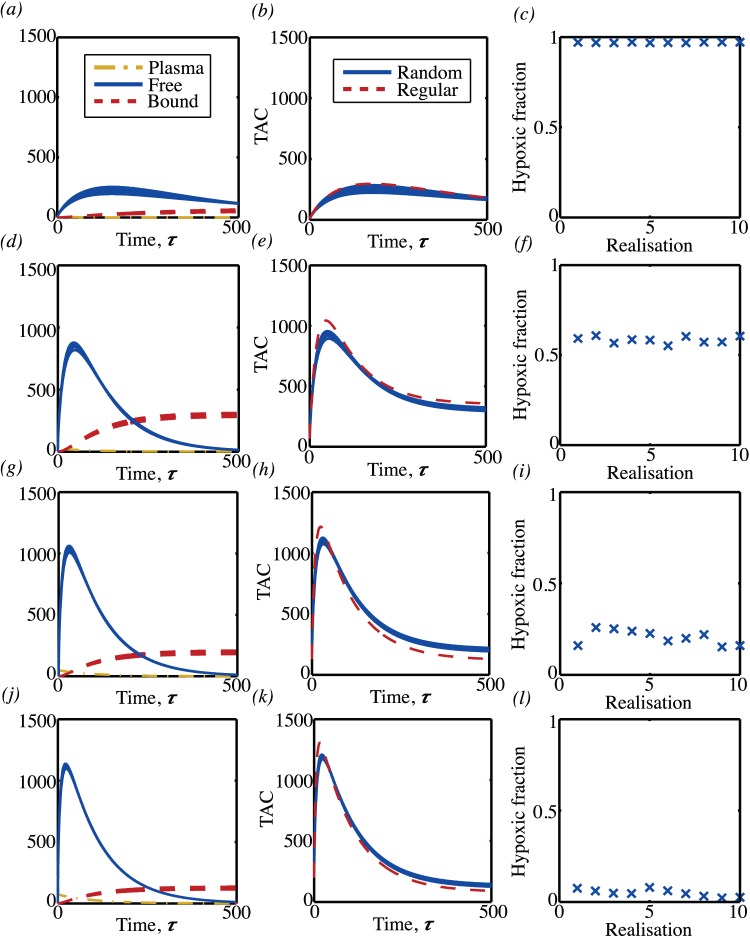
Mean levels of free and bound tracer and TACs computed from the PDE model. Each row corresponds to a different microvessel density (5, 50, 100 and 150 per mm^2^ respectively). The first column shows the contribution to the TAC from the tracer in the blood plasma and the free and bound tracer in the tissue. Ten different random vessel distributions were considered, so ten different sets of curves are shown for each contribution. The central column shows the TACs that result from the ten different random vessel arrangements (solid line) and the TAC as computed from a regular arrangement of vessels (dashed line). The final column shows the hypoxic fraction for each of the different random realisations.

In section 1 it was seen that the distribution of oxygen can be considered as a superposition of the oxygen distribution from single vessels for low enough vessel densities, or equivalently that the oxygen distribution from a regular grid of vessels and that for a random arrangement of vessels give similar oxygenation levels below a vessel density of about 50 for a scaled partial pressure of 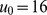 (equating to a partial pressure of 40mmHg if typical parameter values are used). Below, the analogous behaviour is considered for the TAC that results from different microvessel density distributions. For each microvessel density both random and regular vessel distributions are considered. Having specified an arrangement of vessels, the oxygen map is first calculated by solving equations (21). One example of the resulting oxygen map is shown in figure 12 (a). The tracer equations (22) are then solved as a function of time with the plasma input function shown in figure 12(b). The resulting maps for the free and bound tracer at a sequence of points in time are shown in figure 12 (c),(e),(g) and (d),(f),(h) respectively. In figure 12(b) and (c) it is seen how the initial phase is diffusion dominated, with tracer only occuring close to the blood vessels and the bound tracer at a rather lower level than the free tracer. Over time, as seen in (d) and (e) and then in (f) and (g) the free tracer continues to diffuse and is also gradually converted to bound tracer, with highest levels of bound tracer occuring in the hypoxic ‘rings’ that form around blood vessels. These rings have a maximum value at a distance of around five non-dimensional units and are the two-dimensional manifestation of the maximum seen in the tracer profile in figure 10(c). The corresponding TAC for this square of tissue was then calculated by combining the plasma input function, and the free and bound tracer using equation 24. The resulting TACs for different vessel densities are shown in figure 13. At the lowest vessel density of five vessels per mm^2^, as shown in the top row, a regular arrangement of vessels would be indistinguishable from a random arrangement of vessels. More surprisingly, even at high vessel densities, the differences between the random arrangement of vessels and the regular arrangements are rather subtle. This suggests that it is in fact not the random nature of the vessel distribution that is most critical, on the scale of a millimetre.

#### 2.4 Comparison with compartment models

Having computed the oxygen map and the resulting TAC, one can ask to what extent a compartment model can extract parameters such as the mean level of oxygenation. Previous authors have compared both compartment models and partial differential equation models with real experimental data. The advantage of trying to fit a compartment model with ‘experimental data’ generated from a partial differential equation is that one has much greater knowledge and control over the the exact parameter values that are used. If fitting cannot work in this idealised scenario, then it has little hope in the real world.

In order to compare the behaviour of compartment models with a model that includes diffusion of the tracer and the spatial dependence of the oxygen within the tissue we consider the compartment model constructed by Thorwarth *et al* [7] and used in [5], [8], [10]. This model considers three compartments, one for the tracer in the blood, one for the free tracer and one for the bound tracer. The tracer in the blood is modelled by equation (20), the remaining two compartments are modelled by the coupled ordinary differential equations,
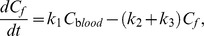


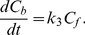
(25)


Here, 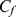 represents the free tracer, 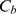 represents the bound tracer. The constants 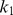 and 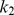 represent the rate at which tracer enters/leaves the free compartment and is related to the permeability of the vessels to the tracer. The constant 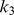 is the net binding rate of the tracer in the tissue and is related to the level of oxygenation of the tissue. Non-dimensionalising by letting 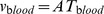, 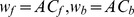 and 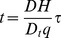 leads to the equations
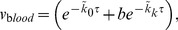
(26)

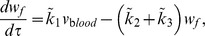


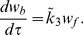
(27)


Initially there is no free or bound tracer so that 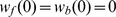, leading to the analytical solution to equations (27)
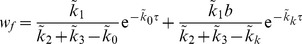
(28)

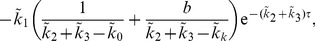
(29)

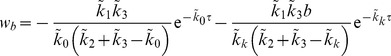
(30)

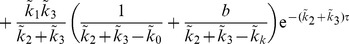
(31)

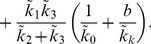
(32)


**Figure 14:**
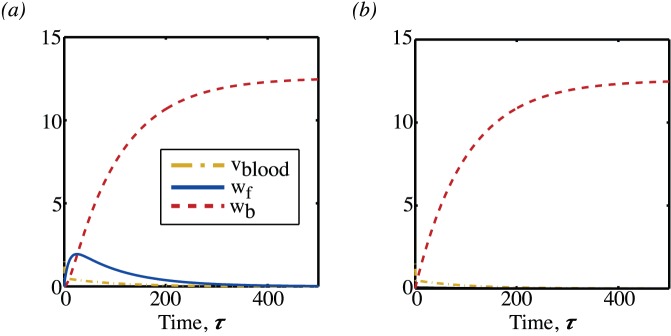
Plasma input function and free and bound tracer concentrations computed from the compartment model. The parameters 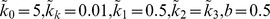 are used in both cases and in (a) 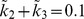, (b) 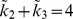.

Typical time tracers of  and 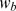 are shown in figure 14. There are three time scales that are important here corresponding to the three different rates that appear in the exponential terms. Typically 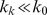 and 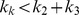, but 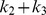 can be either greater or less than 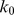 depending on the oxygenation level of the tissue. It is the two timescales 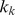 and 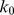 that are relevant for 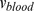, and the fact that 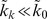 is seen by the very rapid decline in 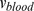 in the first few time units followed by a much slower decline thereafter. For the free tracer, although all three timescales appear in the solution, it is the influence of 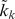 and 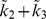 that are most clearly seen in figure 14. The concentration of free tracer first increases then decreases over time as tracer first diffuses from the blood into the free compartment and then leaves to become bound. However, the position and height of the consequent maximum in the free tracer depends on how fast the free tracer is converted to bound tracer relative to the dispersal of tracer around the body as is shown by the two cases in figure 14. In 14(b) 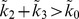, and as soon as the tracer enters the free compartment it is converted to bound tracer so the amount of free tracer remains low.

The TAC consists of a signal with different weightings of the three components, 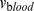, 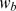 and 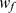. Fitting of the weights along with the rate constant 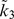 are used to give some idea if tissue is hypoxic or not: hypoxic tissue should have a relatively high value for 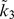 and more bound tracer than normal tissue
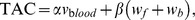


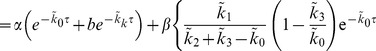


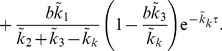
(33)


**Figure 15:**
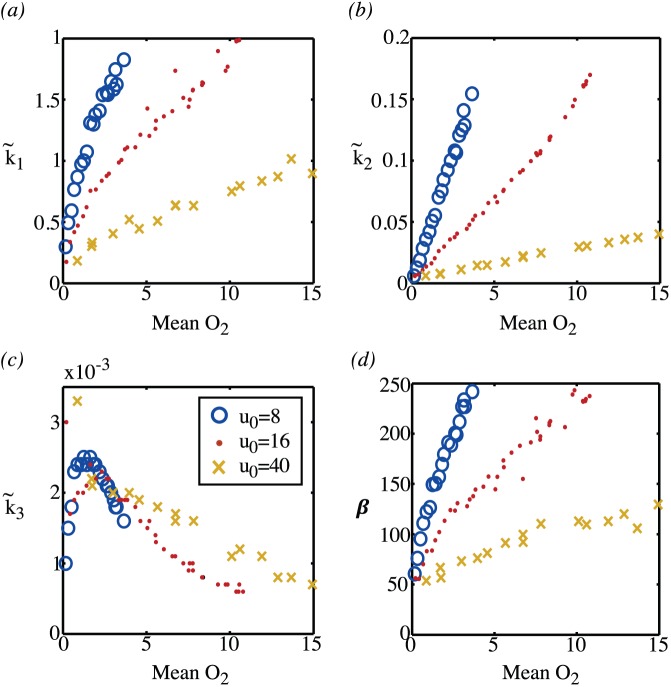
Fitted parameter values. The fitted values of (a) 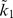, (b) 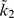, (c) 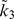 and (d) 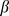 as a function of the mean oxygen level of the tissue. For the computation of each point, first the MVD is fixed. The oxygen map is then computed from equation (21) and the mean oxygen level of the tissue determined. The TAC from the PDE is then constructed by solving equations (22) and computing the expression given in (24). Finally, values of 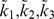 and 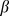 are determined by fitting the TAC from the PDE to the compartment model TAC, equation (33). The circles, points and crosses are calculations for different vessel partial pressures: circles represent calculations with 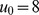; points represent calculations with 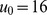, and crosses represent calculations with 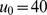.

**Figure 16:**
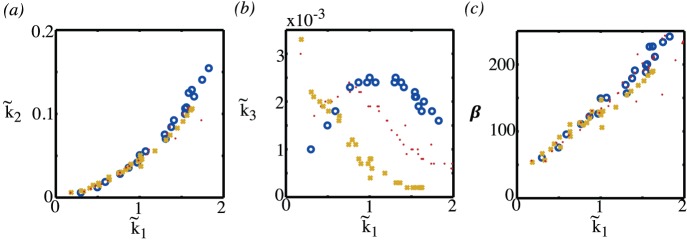
Correlation of 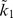 with (a) 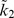, (b) 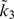
and (c) 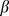. The circles are computations with a vessel partial pressure 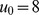; the points are for 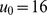, and the crosses are for 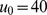.

In each case, we first compute a TAC by solving the partial differential equation model for a particular microvessel density. This computed TAC is then fitted to the formula for the TAC given by (33). We assume that the plasma input parameters, 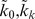 and 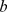, are known and fit for 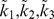 and the weights 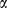 and 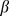. A sequence of calculations for increasing microvessel density was carried out, for each of three vessel partial pressures–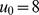, 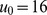 and 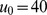 respectively. The results are summarised in figure 15 and figure 16. The parameters 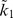 and 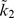 in the compartment model are the rates at which tracer enters and leaves the free tracer compartment. As can be seen from figure 15, the values of this parameter are dependent on both the vessel partial pressure and the mean oxygenation level–or equivalently the microvessel density. The parameter 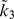, as shown in figure 15(c) is the rate at which oxygen binds to the tissue, and here the nonlinear relationship between the amount of oxygen and the mean value of oxygen is apparent with a binding rate that is highest for hypoxic tissue and low both for very low levels of oxygen, where tissue is predominantly necrotic, and low for high values of oxygen. The parameters 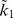, 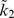 and 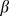 are strongly correlated with each other, as demonstrated in figure 16. Consequently, without knowing the vessel partial pressure it is not possible to deduce information about the mean oxygen levels or, equivalently, the microvessel density from these parameters alone. Values for the parameter 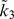 do give a clear indication of hypoxia, with the maximum value of 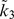 occuring for a non-dimensional mean oxygen level of around 1 (corresponding to 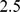 mmHg). Low values of 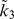 can occur for one of two reasons, either because tissue is necrotic or because tissue is normoxic. The difference between these two cases can be deduced by considering both 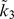 and 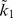: normoxic tissue would have a low value of 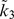 and a high value of 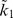 wherease necrotic tissue would have a low value of 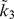 and a low value of 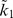.

**Figure 17:**
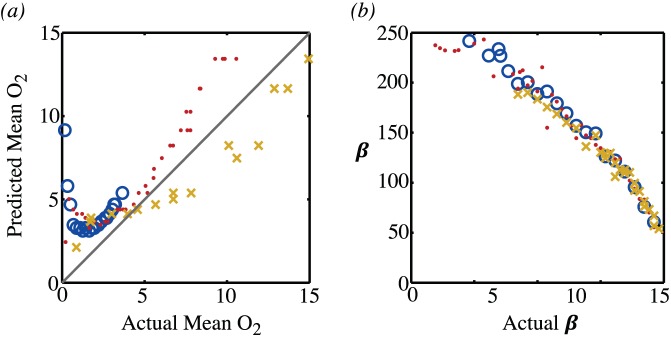
Predictions of the compartment model. (a) The value of the mean oxygen level as predicted by fitting the compartment model to the TAC that is computed from the partial differential equation versus the actual mean oxygen level. (b) The predicted value of the parameter 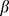 versus the actual value. The circles represent computations with a vessel partial pressure of 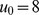; the dots represent computations with 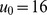 and the crosses represent computations with 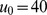.

The parameter 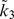 in the compartment model represents the binding rate. This rate is dependent on the mean oxygenation level in the free tracer compartment and should be directly related to the binding rate in the partial differential equation model given in equation (22),
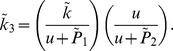
By assuming that the parameters 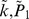 and 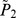 are known, one can invert this relationship and examine to what extent the value of 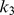 given by fitting the compartment model correlates to the actual mean value of oxygen given by the partial differential equation calculation, see figure 17.

## Discussion

Modelling the distribution of tracer in the body is a difficult task. There are a number of different levels of uncertainty and inaccuracy. Firstly, in writing down a mathematical model various modelling assumptions are made as to which processes may be neglected and which cannot. Secondly, in most models there are parameters which have to be determined. The value of these parameters can affect both the qualitative and quantitative behaviour of the model. Finally, there are computational errors that are introduced when numerical methods are used to solve equations. If a mathematical model is to be of use, these different types of error need to be quantified and, ideally, an estimate of the uncertainty of any result made.

In this paper we have sought to quantify the effect of some of these sorts of error for the particular problem of oxygen diffusing in a (two-dimensional) piece of tissue and the consequent tracer dynamics. We have addressed two particular modelling issues: firstly the consequence of using different kinds of boundary condition to describe the flow of oxygen from blood vessel to tissue and secondly the extent to which compartment models can be used to describe tracer concentration in tissue where the oxygen distribution is inhomogeneous. For typical vessel permeabilities and partial pressures for tumour tissue, we have found that using a Dirichlet type boundary would typically result in an overestimate of the amount of oxygen by a factor of two, suggesting that either mixed, or the source method should be used. The fact that the source method gives good results, is significant as this is a method that is likely to be easier to implement in three space dimensions than modelling blood vessels as discrete entities with flux boundary conditions. The second modelling assumption that we have investigated is to what extent the heterogeneous nature of the vascular supply is important/detectable by a TAC that averages over a region of a square millimetre. In fact, the actual distribution of the vessels does not significantly affect the form of the TAC: TACs from both regular and randomly arranged blood vessels were strikingly similar with the qualitative and quantitative features much more strongly dependent on the partial pressure of the vessels and their number. In part, this is because after the first few minutes, although one can still see the after-effects of the position of the blood vessels in the spatial distribution of the tracer, as shown in figure 12, the actual magnitude of the variation at any point in time is relatively small. This is because typical diffusion times for tracers, 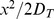, are an order of magnitude shorter than typical times associated with the binding for tracers, as given by 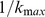. In real tissue, the vessels are not only highly heterogeneous in their position, but also in their size, vessel partial pressure and vessel blood flow rates. However, the difference in timescales between the diffusion and the chemical kinetics suggests that this heterogeneity is averaged out by the diffusion process and is not detectable on the timescale of the chemical kinetics. The consequence is that fitting a TAC to a partial differential equation model including the full heterogeneity in the distribution and the characteristics of the vessels will result in essentially the same prediction for mean oxygen partial pressure as fitting to a compartment model. While the partial differential equation models that include vascular structures are valuable for allowing the investigation of how changes to the underlying physiological parameters affect the results, this suggests that compartment models will be sufficient in a clinical setting.

One of the difficulties in comparing with experiment is that some of the parameters are hard to measure. The sensitivity results of section 1.3 showed that a 10% error in the scaled permeability 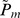 or the scaled partial pressure 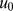 will result in a similar error in the oxygenated area. Similarly the value of the oxygen threshold used to define the oxygenated area, 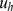, has a significant effect on the results.

Finally we note that one source of error is the computational error: for the sake of computational time, many authors have used a single point to represent a vessel. As we have seen, this in itself can introduce an error of approximately 30%.

## Supporting Information

Table S1 **Measured values for the physical parameters.**
(PDF)

Table S2 **Parameter ranges for non-dimensional quantities.**
(PDF)
