# Supplementary material for: Modelling and Detecting Tumour Oxygenation Levels
Source: PLoS One. 2012 Jun 28;7(6):e38597. doi: 10.1371/journal.pone.0038597 (PMC3386285; doi:10.1371/journal.pone.0038597)
Supplement: Table S1 — Measured values for the physical parameters. (PDF) [file pone.0038597.s001.pdf]

Table S1: Measured values for the physical parameters

Parameter		Value	Source
Radius, R		6μm	[13]
		7μm	[12]
Diffusivity of oxygen in tissue, D		0.002mm^{2}s^{-1}	[12]
		4.2×10^{-10}cm$^3 O_2$/cm/s/mmHg.	[20]
$K_{\max}$		15mmHg s^{-1}	[13]
		$2 - 16$mmHg s^{-1}	[12]
P_{50}		2.5mmHg	[13]
Partial pressure in vessel, P_v		$20 - 100$mmHg	
		40mmHg	[9]
		$30 - 80$mmHg	[20]
Permeability of capillaries to oxygen, P_m		0.3 mm s^{-1}	
		0.06 mm s^{-1}	[9]
Vessel density		≈ 60 mm^{-2}	[9, 13]
Hypoxic level, P_h		5 mmHg	[9]
		$1 - 14$ μM	[12]
		$2.5, 5$ mmHg	[13]
Diffusivity of tracer in tissue, D_T		5.5×10^{-5} mm^2 s^{-1}	[10]
Permeability of capillaries to tracer, P_T		$0.024 - 0.094$ mm s^{-1}	[9]
Tracer binding constants	k_{max}	1.7×10^{-4} s^{-1}	[10]
		2.4×10^{-3} s^{-1}	[6]
		8.0×10^{-4} s^{-1}	[21]
	P_1	$0.8 - 1.5$ mmHg	[10]
		2710 ppm	[6]
		1.8 mmHg	[21]
	P_2	0.6 mmHg	[21]
Blood input parameters	A	$8000 - 12000$	[21]
	k_0	$0.13 - 0.22$	[21]
	k_k	$0.001 - 0.002$	[21]
	b	$0.1 - 1.67$	[21]
