# Supplementary material for: Modelling and Detecting Tumour Oxygenation Levels
Source: PLoS One. 2012 Jun 28;7(6):e38597. doi: 10.1371/journal.pone.0038597 (PMC3386285; doi:10.1371/journal.pone.0038597)
Supplement: Table S2 — Parameter ranges for non-dimensional quantities. (PDF) [file pone.0038597.s002.pdf]

Table S2: Parameter ranges for non-dimensional quantities

Parameter	Range
$\widetilde{R} = \sqrt{\frac{q}{Dh}}R$	0.55
$\widetilde{P}_m = \sqrt{\frac{h}{Dq}}P_m$	$0.55 - 2.75$
$u_0 = \frac{1}{h}P_v$	$8 - 40$
$\widetilde{P}_T = \sqrt{\frac{Dh}{D_T^2 q}}P_T$	$2 - 60$
$\widetilde{k}_{max} = \frac{Dh}{D_T q}k_{max}$	$0.001 - 0.1$
$\widetilde{P}_1 = \frac{1}{h}P_1$	0.6
$\widetilde{P}_2 = \frac{1}{h}P_2$	0.24
$\widetilde{k}_0 = \frac{Dh}{D_T q}k_0$	$0.7 - 10$
$\widetilde{k}_k = \frac{Dh}{D_T q}k_k$	$0.006 - 0.09$
